# Secondary breast lymphoma diagnosed by vacuum-assisted breast biopsy: a case report

**DOI:** 10.1186/1752-1947-1-113

**Published:** 2007-10-23

**Authors:** Flora Zagouri, Theodoros N Sergentanis, Afroditi Nonni, Dimitra Koulocheri, Philip Domeyer, Dimitrios Dardamanis, Nikolaos V Michalopoulos, Nikolaos Pararas, Antonia Gounaris, George C Zografos

**Affiliations:** 1 Breast Unit, 1st Department of Propaedeutic Surgery, Hippokratio Hospital, University of Athens; 114, Vas Sofias Ave, Athens 116 27, Greece; 2 Research Center, Hellenic Anticancer Institute; 11, Valtetsiou str., Athens106 80, Greece

## Abstract

**Introduction:**

Breast lymphoma, either as a manifestation of primary extranodal disease or as secondary involvement, is a rare malignancy, and its diagnosis, prognosis, and treatment have not been clearly defined. On the other hand, Vacuum-assisted breast biopsy (VABB) is a minimally invasive technique with ever-growing use for the diagnosis of mammographically detected, non-palpable breast lesions.

**Case presentation:**

A symptom-free, 56-year-old woman presented with a non-palpable BI-RADS 4B lesion without microcalcifications. She had a positive family history for breast cancer and a history of atypical ductal hyperplasia in the ipsilateral breast four years ago. She reported having been treated for non-Hodgkin lymphoma 12 years ago. With the suspicion of breast cancer, mammographically guided VABB with 11-gauge probe (on the stereotactic Fisher's table) was performed. VABB made the diagnosis of a non-Hodgkin, grade II, B-cell germinal-center lymphoma. VABB yielded enough tissue for immunohistochemistry/WHO classification.

**Conclusion:**

This is the first case in the literature demonstrating the successful diagnosis of breast lymphoma by VABB, irrespectively of the level of clinical suspicion. It should be stressed that VABB was able to yield enough tissue for WHO classification. In general, lymphoma should never be omitted in the differential diagnosis, since no pathognomonic radiologic findings exist for its diagnosis.

## Introduction

Breast lymphoma, either as a manifestation of primary extranodal disease [1,2] or as secondary involvement [3,4], is a rare malignancy and its diagnosis, prognosis, and treatment have not been clearly defined. Relatively small groups of patients are reported in the literature. The reported incidence of primary breast lymphoma ranges from 0.04% to 0.5% of all breast malignancies [1,2]. Secondary breast lymphoma is less well studied than primary lymphoma in the literature and it is also rare, with a reported incidence of 0.07% [3,4]. The diagnosis of breast lymphoma is usually performed by fine needle aspiration cytology, with a reported sensitivity ranging from 83% to 100% [3,5].

Vacuum-assisted breast biopsy (VABB) is a minimally invasive technique with ever-growing use for the diagnosis of mammographically detected, non-palpable breast lesions. The sensitivity, specificity and fast performance of the method have contributed to its gradual establishment in the biopsy of suspicious breast lesions [6]. In this study, VABB (11 G) was mammographically guided and conducted on the stereotactic Fisher's table.

VABB is effective in the assessment of breast lesions both with and without microcalcifications [6]. Exhibiting a very low rate of false negative results and capable of excising a great amount of tissue, VABB has been proven to have superior sensitivity than fine needle aspiration and core biopsy in breast cancer diagnosis [7,8].

We present the first case in the literature of a secondary breast lymphoma diagnosed by VABB, despite the absence of strong clinical suspicion.

## Case presentation

A 56-year-old woman came to our Breast Unit for her annual follow-up. A newly developed, non-palpable solid lesion of diameter equal to 0.8 cm was present in the upper outer quadrant of the left breast. The lesion did not contain microcalcifications, and axillary lymph nodes of small size were detected on the mammogram. The ultrasound examination was negative.

From the personal history, 12 years ago, a low-grade, stage I, Non-Hodgkin lymphoma confined to the lymph nodes of the neck was diagnosed and treated with radiation therapy. The patient was symptom-free, and able to function normally in her everyday life.

The woman had risk factors for breast cancer: positive family history for breast cancer (mother with postmenopausal breast carcinoma), a history of atypical ductal hyperplasia (ADH) 4 years ago, diagnosed by excisional biopsy under local anesthesia for a palpable lesion. Her BMI was equal to 25, and she was a housewife. The age at menarche was 15 years old and the age at drug-induced menopause was 44 years. The patient has had two induced abortions and three full-term pregnancies. The duration of lactation for all three full-term pregnancies was 15 months. There was no family history for ovarian and prostate cancer. There was no history of intake of estrogen.

**Figure 1:**
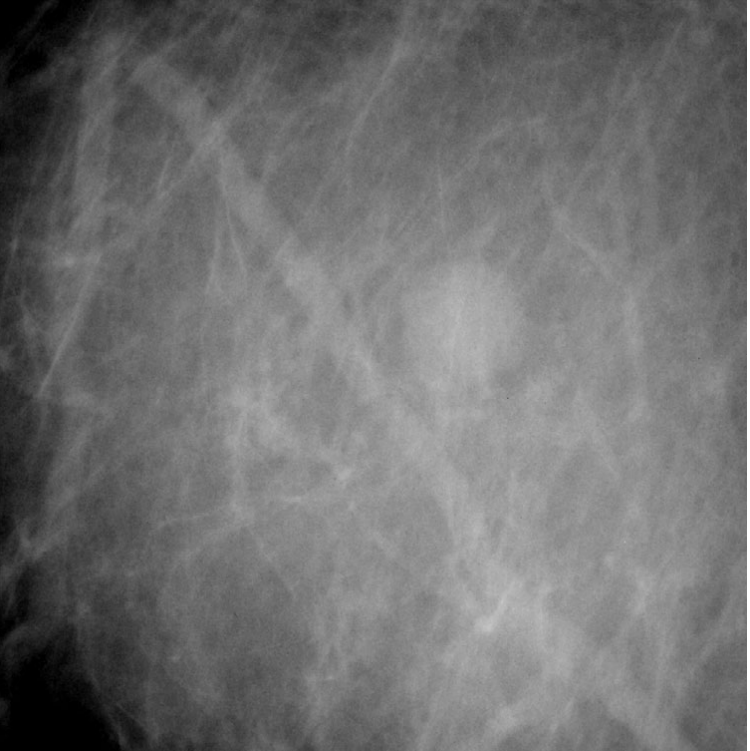
The suspicious lesion on the screen (Fischer workstation, VABB device).

**Figure 2:**
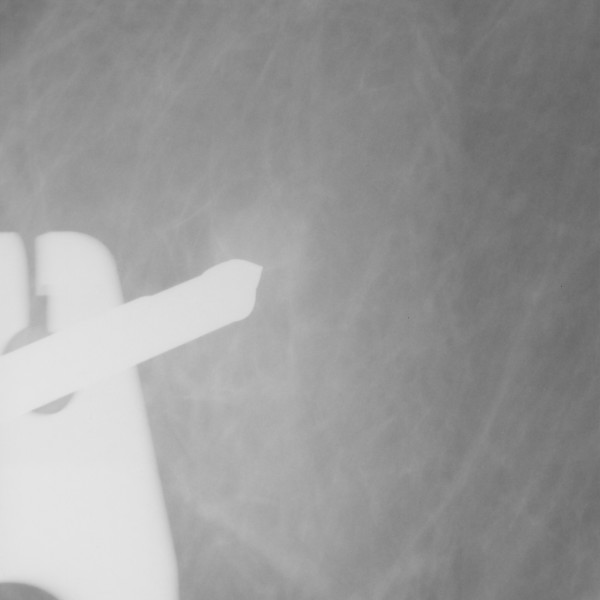
The lesion at the onset of sampling.

**Figure 3:**
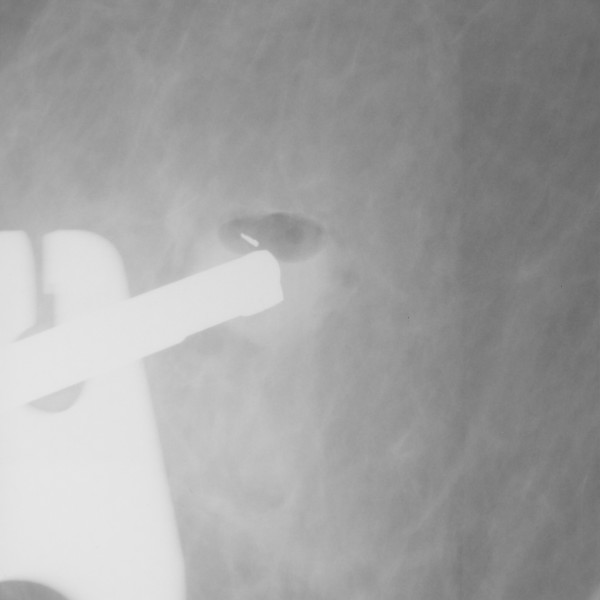
The lesion after sampling.

The radiologist of our Unit characterized the suspicious lesion as BI-RADS 4B (lesion with an intermediate suspicion of malignancy) and a VABB was scheduled. Lymphoma was not suspected at presentation. VABB was performed on a digital prone table (Mammotest, Fischer Imaging, Denver, CO, USA) using 11-gauge Mammotome vacuum probes, under local anesthesia (Figures 1, 2). Using one main target-offset and one offset inside the solid lesion, 24 cores were excised from the suspicious lesion (Figures 1, 2, 3). The length of the cores varied between 0.5 and 2.8 cm. A clip marker was placed after biopsy and a mammogram to the affected breast confirmed the excision of cores in the lesion.

**Figure 4:**
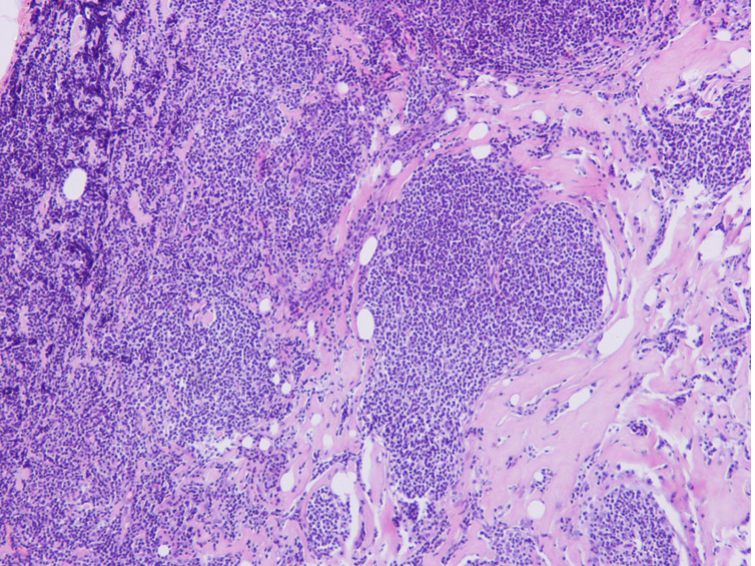
Invasion of the breast parenchyma by the nodular non-Hodgkin B-cell lymphoma (H+E × 100).

**Figure 5:**
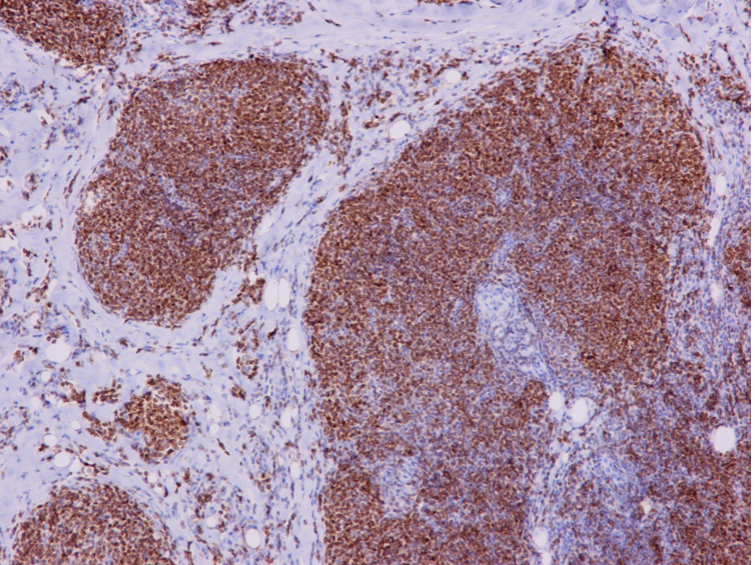
Immunoreaction of the lymphoid cells to bcl-2 protein (× 200).

According to the pathological examination, the lesion was a non-Hodgkin, grade II, germinal-center lymphoma of B-cell origin and exhibited a nodular pattern (> 75%) (Figure 4). More specifically, the nodules were formed by cells with morphology of centroblasts and centrocytes; the number of centroblasts did not exceed 15 per optical field (×40). The stroma exhibited hyalinization where the malignant lymphoid tissue was present. The malignant lymphoid cells were occasionally present within the fatty breast tissue, and a few entrapped mammary ducts were recognized within the sclerotic stroma. Immunohistochemically, the lymphoid cells were positive for CD20, bcl-2 (Figure 5) and CD10, whereas some CD10-positive cells were present outside the nodules. There was indication of kappa chain clonality. The Ki-67 proliferation marker was positive in 10% of cells.

Subsequently, after second contact with the patient, and the establishment of the diagnosis, the patient disclosed that she had stopped her hematological follow-up 6 years ago. She was then referred to the hematologists for further evaluation and treatment.

## Discussion

The present case of breast NHL presented as a lesion without microcalcifications. Indeed, breast NHL most commonly presents as a solitary, uncalcified mass. However, it has been demonstrated that no pathognomonic radiologic findings exist for the differential diagnosis between breast lymphoma and carcinoma [9]. More interestingly, in the context of non-palpable mammographic lesions, such as the present one, there is scarcity of data on issues regarding the differential diagnosis.

Although the distinction between lymphoma and carcinoma seems demanding, accurate differential diagnosis before treatment is necessary, irrespective of the level of suspicion. That is because the treatment of lymphoma differs radically from that of carcinoma [3]. In the hypothetical scenario that a lymphoma is missed at presentation, lumpectomy or mastectomy (unnecessary treatments for lymphoma) might be performed.

In the present case, the existence of two significant risk factors (a diagnosis of ADH in the past and positive family history) reinforced the clinical suspicion for breast cancer, which is otherwise the prevailing one in the everyday context of a Breast Unit. More specifically, the above two risk factors have been associated with a relative risk equal to 9.7 in the literature [10].

Fine-needle aspiration (FNA) is the established diagnostic procedure for the diagnosis of breast lymphomas [5,11]. FNA, however, bears certain limitations: in terms of breast lesions in general, FNA has been proven to yield insufficient diagnostic material [12]. More specifically and regarding breast lymphoma, FNA occasionally fails to establish the WHO classification in secondary lesions and, more frequently, in primary lesions [11]; this is of particular importance, since the WHO classification is crucial for the long-term management and prognosis. Given the above inherent difficulties, the role of confirmatory core biopsy has been discussed [3].

In the present case, VABB successfully made the diagnosis and provided adequate tissue for the evaluation of the molecular markers/WHO classification. Many reasons account for this: the excision of a significant percentage of the non-palpable solid lesion, the accurate targeting in the stereotactic-digital setting, the multiple cores via a single needle insertion and the large amount of tissue obtained. Indeed, it is standard practice in our Center to obtain more than 24 cores in each lesion, irrespectively of the degree of clinical suspicion; this is above the majority of existing studies (cf. [13]).

Interestingly enough, breast lymphoma has been shown to escape diagnosis by nonimage-guided core needle biopsy (14 G), as shown by a recent series [14]. However, when ultrasound-guided, core biopsy has been once able to detect breast lymphoma [15]. Our described case may seem thus rational in the continuum of recently developed breast techniques; bearing the features explained above, the most recently evolved technique of VABB might be advantageous with respect to breast lymphoma. It is tempting to speculate that the obtention of a relatively great volume by VABB might have comparable performance to that of excisional biopsy *vis-à-vis *breast lymphoma.

## Conclusion

This is the first reported case of breast lymphoma diagnosed by VABB in the international literature. Despite the lack of clinical suspicion, the lymphoma did not escape VABB, and VABB yielded adequate tissue for WHO classification. As the experience with VABB accumulates worldwide, the performance of the method remains to be accurately assessed in the future.

## Competing interests

The author(s) declare that they have no competing interests.

## Authors' contributions

FZ conceived the idea of the study and wrote the manuscript. TNS interpreted the case findings with respect to international literature and wrote the manuscript. AN made the pathological diagnosis and the immunohistochemical evaluation of molecular markers. DK assigned the BI-RADS category and assisted in VABB. PD performed review of the literature and assisted in VABB. DD, NVM, NP performed VABB and participated in the evaluation of the case findings. AG revised the manuscript for important intellectual content. GCZ supervised and performed VABB, interpreted the findings, revised critically the manuscript for important intellectual content and gave final approval of the version to be published.

## Consent

Written informed patient consent was obtained for publication.
